# Incidental Bilateral Ductal Carcinoma In Situ (DCIS) in Excisional Surgery for Gynecomastia

**DOI:** 10.7759/cureus.63974

**Published:** 2024-07-06

**Authors:** Ariel Toomey, Michele Champigny, Jefrey Fishman, Maria Miglio

**Affiliations:** 1 Plastic & Reconstructive Surgery, Corewell Health William Beaumont University Hospital, Royal Oak, USA; 2 Plastic & Reconstructive Surgery, Michigan State University College of Osteopathic Medicine, Royal Oak, USA

**Keywords:** plastic and reconstructive surgery, body contouring after weight loss, dcis in male gynecomastia, male breast cancer, bilateral gynecomastia, ductal carcinoma in situ (dcis)

## Abstract

Male breast cancer is a rare disease, and it is important to have a high index of suspicion in patients presenting with breast symptoms, such as a breast mass or nipple discharge. Most male patients who are diagnosed with breast cancer present with breast complaints and/or a strong family history of cancer. Here, we will present a 47-year-old male patient who was diagnosed with bilateral ductal carcinoma in situ during a routine gynecomastia surgery after massive weight loss. This case demonstrates the importance of sending breast tissue specimens for pathology, especially in a male patient.

## Introduction

Breast cancer in males is extremely rare and accounts for <1% of all cases of breast carcinoma and <1% of all male malignancies [1,2]. There are many distinct differences between breast cancer presentations in males and females. In males, breast cancer tends to present later in the course of the disease, at a higher clinical stage, and with more frequent lymph node metastases [2]. There also seems to be a less favorable outcome in males with breast cancer than in females with the same disease [2]. However, there does not seem to be a significant prognostic difference between male and female breast cancer of the same stage [1]. 

Ductal carcinoma in situ (DCIS) is a premalignant neoplasia pathologically confined to the lumen of mammary ducts [3]. It accounts for over 20% of all new breast cancer diagnoses in women in the US; conversely, it only accounts for 0.1% of new breast cancer diagnoses in men [2,3]. If the neoplasm penetrates through the basement membrane, it is considered invasive ductal carcinoma [3]. Despite the extensive literature on women, very little is known about DCIS in males. Most cases of DCIS in men are included with a larger series of invasive breast cancer; therefore, it is hard to differentiate whether they are purely in situ lesions or involved with an invasive component.

## Case presentation

A 47-year-old male presented to the office on October 13, 2022, for evaluation following massive weight loss. At the time, he had lost approximately 130 pounds participating in a weight loss program of diet modification and exercise. His three biggest areas of concern were his chest, abdomen, and thighs. His weight had been stable for at least six months. His medical history includes obstructive sleep apnea, hypertension, and gastroesophageal reflux, for which he takes amlodipine 5 milligrams (mg) daily, losartan-hydrochlorothiazide 50/12.5 mg daily, and omeprazole 40 mg daily. His social history includes previous cigarette smoking (quit 10 years prior), current vaping, and about two alcoholic drinks per week. Family history includes diabetes, hypertension, and obesity. He had no family history of cancers, myocardial infarction, or cerebrovascular accident. He has no pertinent surgical history.

**Figure 1:**
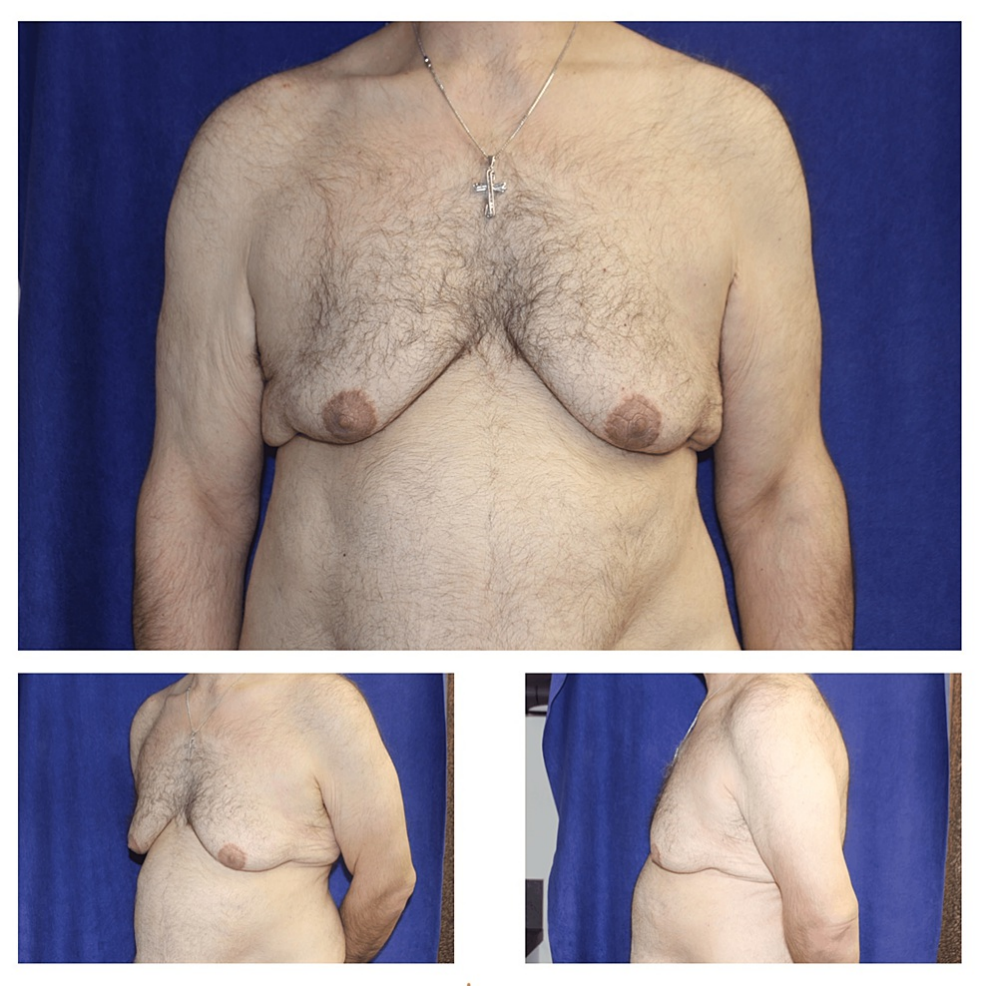
Preoperative pictures demonstrating gynecomastia in the patient

On exam, the patient was noted to have significant gynecomastia with grade II-III ptosis with excess cutaneous tissue and redundancy extending through the axillary tail region onto the lateral chest wall and extending onto the lateral chest wall posteriorly toward the back (Figure 1). There were no breast masses, no nipple discharge, no skin changes, and no axillary adenopathy. An exam of the abdomen revealed significant ptosis and redundancy with a hanging ptotic abdominal pannus. In addition, the mons area demonstrated rather significant ptosis. There were no abdominal herniations appreciated. The abdominal redundancy extended well into the waist and flank areas. Exam of the thighs revealed significant redundancy with ptosis, predominantly the upper medial thigh region, as well as superficial varicosities, more so on the left than on the right. 

Details of each procedure and risks, benefits, and alternatives were reviewed. The recommended first stage of treatment proposed was extended abdominoplasty with monsplasty and excision of bilateral gynecomastia with free nipple grafting and modified upper body lift. The patient underwent the proposed procedures on January 11, 2023. He was admitted to extended recovery post-operatively, had an uneventful postoperative course, and was discharged home on January 12, 2023. 

The patient was directed to the emergency department on postoperative day five for complaints of right thigh pain. He had an ultrasound that showed superficial vein thrombosis but no deep vein thrombosis and was prescribed Motrin 800 mg every six hours, daily aspirin 81 mg, and warm compresses and recommended to keep his regularly scheduled post-operative appointment. The patient’s first post-operative appointment was on January 17, 2023, and he was healing well. His chest drains were removed, and all but one of the abdominal drains were removed. He was told to follow up in one week. At his second post-operative appointment, the abdominal drain was maintained due to increased output and was discontinued the following week. 

The pathology from the gynecomastia excision resulted in bilateral DCIS, grade 1, with no evidence of invasive carcinoma, and negative margins. Bilateral specimens were estrogen and progesterone receptor-positive. The patient was referred to a breast surgeon and medical oncologist, and no further treatment was recommended. No post-operative photos have been obtained. 

## Discussion

DCIS is a premalignant lesion of the breast composed of neoplastic glandular epithelium confined to the lumen of the ducts that have not yet invaded the basement membrane [3]. DCIS in men is extremely rare, accounting for only about 10% of male breast cancers when involved with an invasive component [2]. Pure DCIS is even more sporadic accounting for <5% of all diagnosed male breast cancers [2].

Because of the rarity of this disease, it is essential to recognize the key signs and symptoms that may lead to the diagnosis. The most notable symptoms of male DCIS are a subareolar mass with slow growth and serosanguineous nipple discharge [1]. Other symptoms to be aware of include gynecomastia and mastitis, but these are rare [1]. DCIS more commonly presents in males over 40 years old [4]. Aside from signs and symptoms, patient and family history are important to collect. There is a possible link between familial breast carcinoma and pure DCIS in males [5].

The median duration of symptoms for pure DCIS has been reported around two months, but those with invasive carcinoma and DCIS have a longer duration of symptoms at around six months [6]. Due to the anatomy of the male breast and that the lesions are usually central and subareolar, the nipple is commonly involved [6]. In contrast to DCIS in females, radiologically, male DCIS rarely presents with calcifications and only in 10% of cases presents as a cystic nodule [3]. Therefore, cytological evaluation of all male breast lesions is standard for diagnosis [3].

**Figure 2:**
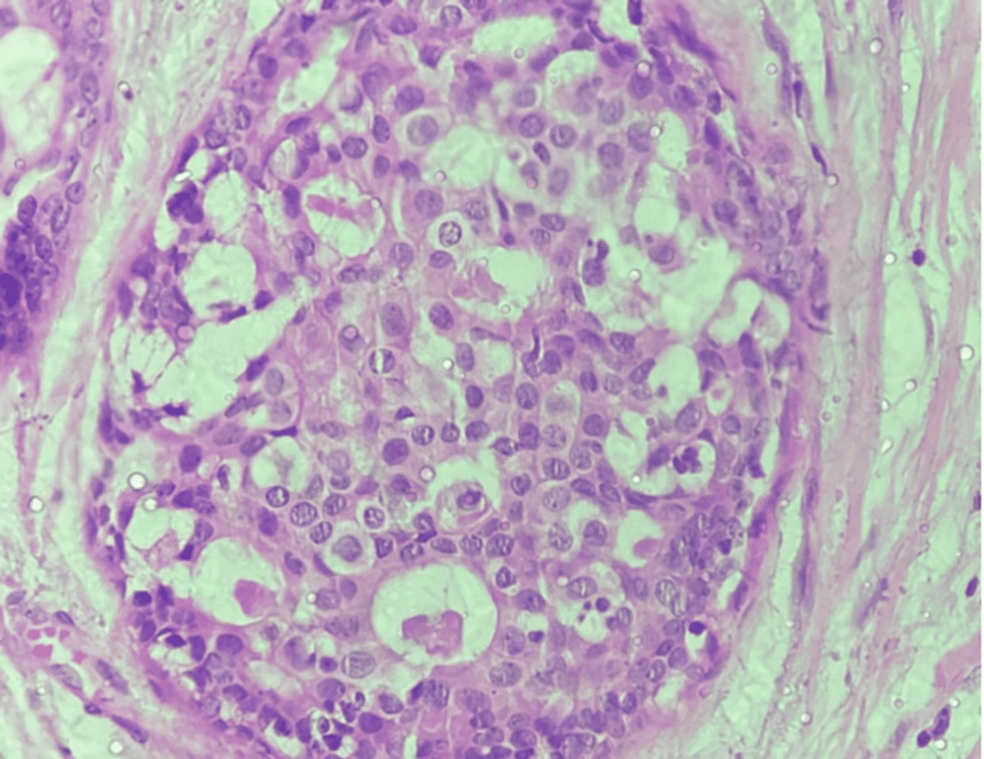
Hematoxylin and eosin stain at 40X shows cribriform-type ductal carcinoma in situ (DCIS). Histopathological examination of DCIS in a post-operative specimen from the Department of Pathology, Vardhman Mahavir Medical College and Safdarjung Hospital, New Delhi [3].

Histologically, male and female DCIS have similar characteristics and patterns. Hittmair et al. [1] looked at 84 cases of pure male DCIS and found that the most common histologic subtype was papillary carcinoma with a superimposed cribriform pattern (74%). An example of this is shown in Figure 2. Most of the lesions in this case study were either low or intermediate-grade, and there have been no reported high-grade pure DCIS cases in the literature [1]. Moreover, in the study by Hittmair et al. [1], most patients of male DCIS were estrogen-receptor and progesterone-receptor positive (65%) and had a low Ki67 (<20%).

Treatment for DCIS in males is mastectomy with sentinel lymph node biopsy, with a margin of at least 2 mm recommended [3,4]. It is important to note that about 26% of patients will be upstaged from DCIS to invasive carcinoma after a final histologic examination [4]. Adjuvant treatments such as endocrine and/or radiation therapy are not usually recommended in male patients with DCIS, although a multidisciplinary approach is strongly encouraged [4].

Prognosis in male patients with pure DCIS is excellent with a 93.3% five-year overall survival rate, which is comparable to females with the same diagnosis [4].

## Conclusions

DCIS in men is an extremely rare disease that offers an excellent prognosis. Very few cases of pure male DCIS (without an invasive component) have been described. Here, we discuss an example of incidentally discovered bilateral DCIS (low grade) after excisional surgery for gynecomastia, which after a multidisciplinary team approach required no further treatment.
